# Nanomaterials for mRNA‐based therapeutics: Challenges and opportunities

**DOI:** 10.1002/btm2.10492

**Published:** 2023-01-29

**Authors:** De‐feng Li, Qi‐song Liu, Mei‐feng Yang, Hao‐ming Xu, Min‐zheng Zhu, Yuan Zhang, Jing Xu, Cheng‐mei Tian, Jun Yao, Li‐sheng Wang, Yu‐jie Liang

**Affiliations:** ^1^ Department of Gastroenterology Shenzhen People's Hospital (the Second Clinical Medical College, Jinan University; the First Affiliated Hospital, Southern University of Science and Technology) Shenzhen Guangdong China; ^2^ National Clinical Research Center for Infectious Diseases Shenzhen Third People's Hospital, Southern University of Science and Technology Shenzhen China; ^3^ Department of Hematology Yantian District People's Hospital Shenzhen Guangdong China; ^4^ Department of Gastroenterology and Hepatology Guangzhou Digestive Disease Center, Guangzhou First People's Hospital, School of Medicine, South China University of Technology Guangzhou China; ^5^ Department of Gastroenterology and Hepatology the Second Affiliated Hospital, School of Medicine, South China University of Technology Guangzhou Guangdong China; ^6^ Department of Medical Administration Huizhou Institute of Occupational Diseases Control and Prevention Huizhou Guangdong China; ^7^ Department of Emergency Shenzhen People's Hospital (the Second Clinical Medical College, Jinan University; the First Affiliated Hospital, Southern University of Science and Technology) Shenzhen Guangdong China; ^8^ Department of Child and Adolescent Psychiatry Shenzhen Kangning Hospital, Shenzhen Mental Health Center Shenzhen China; ^9^ Affiliated Hospital of Jining Medical University, Jining Medical University Jining Shandong China

**Keywords:** Exosomes, Gene editing, Gene therapy, mRNA delivery, mRNA vaccine, Nanocarriers

## Abstract

Messenger RNA (mRNA) holds great potential in developing immunotherapy, protein replacement, and genome editing. In general, mRNA does not have the risk of being incorporated into the host genome and does not need to enter the nucleus for transfection, and it can be expressed even in nondividing cells. Therefore, mRNA‐based therapeutics provide a promising strategy for clinical treatment. However, the efficient and safe delivery of mRNA remains a crucial constraint for the clinical application of mRNA therapeutics. Although the stability and tolerability of mRNA can be enhanced by directly retouching the mRNA structure, there is still an urgent need to improve the delivery of mRNA. Recently, significant progress has been made in nanobiotechnology, providing tools for developing mRNA nanocarriers. Nano‐drug delivery system is directly used for loading, protecting, and releasing mRNA in the biological microenvironment and can be used to stimulate the translation of mRNA to develop effective intervention strategies. In the present review, we summarized the concept of emerging nanomaterials for mRNA delivery and the latest progress in enhancing the function of mRNA, primarily focusing on the role of exosomes in mRNA delivery. Moreover, we outlined its clinical applications so far. Finally, the key obstacles of mRNA nanocarriers are emphasized, and promising strategies to overcome these obstacles are proposed. Collectively, nano‐design materials exert functions for specific mRNA applications, provide new perception for next‐generation nanomaterials, and thus revolution of mRNA technology.

## 1 INTRODUCTION

As intermediate tunneling, messenger ribonucleic acid (mRNA) can transport genetic codes from DNA to ribosomes for protein expression, which has emerged as a highly appealing potential to change the vaccination, protein replacement therapy, and other treatments for human diseases.^1^, ^2^, ^3^, ^4^ The first study of successful mRNA therapeutics in the preclinical application was published in 1990.^5^ In addition, due to the progress of mRNA manufacturing and intercellular delivery strategy, significant development and breakthrough have been made in the mRNA‐based therapy.^6^ In addition, mRNA‐based therapeutics exhibit several advantages over traditional functional biomolecules and therapeutic proteins.^7^ First, mRNA is endowed with perfect safety under physiological conditions because it is not transferred into the nucleus and does not integrate into the host genome. Therefore, mRNA‐based therapeutics are transient, avoiding the potential risk of insertional mutagenesis.^8^, ^9^ Second, the initiation of protein translation is promoted once mRNA just reaches the cytoplasm.^10^ Third, these theranostic mRNAs can achieve stability and controlled release through mRNA structural modification (such as 5′‐Cap, 5′‐UTR, ORF, 3′‐UTR, and 3′‐poly(A) tail) or nanomaterials delivery, such as lipid‐derived nanoparticles (LNPs), polymer‐based nanoparticles, and hybrid nanoparticle, thus improving the pharmacokinetics of nucleic acid drugs.^11^, ^12^, ^13^ Fourth, it is easy to design and manufacture mRNA using chemical synthesis and enzyme synthesis approaches.^14^ Solid‐phase chemical synthesis is generally suitable for the synthesis of short‐chain RNAs (50–100 nt). Moreover, the synthesis yield using this method remains poor and impure with the increase of RNA length.^15^ Recently, enzyme synthesis is a popular method based on RNA polymerase (usually T7) and linear DNA to obtain desired mRNA. However, the initial product of mRNA through this method is composed of targeted mRNA, untargeted mRNA, and oligodexynucleotides, which need to be purified for further application.^16^

Despite its potential advantages, how to efficiently and stably perform intracellular delivery of mRNA is a significant barrier in the clinical application of mRNA as a therapeutic modality.^14^, ^17^, ^18^, ^19^ Additionally, naked mRNA molecules are easily degraded by enzymes and are not easily taken up by the target cells.^20^ Therefore, methods and vehicles for mRNA delivery, such as viral vectors, mechanical transfection, and nonviral vectors, have been developed.^11^, ^21^, ^22^ Viral vectors are very efficiency in mRNA transfection and are applied in clinical trials, such as Kymriah for chimeric antigen receptor (CAR) T immunotherapy on lymphoblastic leukemia.^23^, ^24^ However, viral vectors have disadvantages of potential carcinogenic, high immunogenic, limited gene packaging ability, and low‐volume production.^25^, ^26^ Mechanical transfection, such as injection of naked mRNA in conventional and self‐amplifying forms, is not widely applied due to their extracellular exonucleases, inefficient cell uptake, unsuccessful endosomal release, or potential cytotoxicity.^27^ Nonviral vehicles, including lipid or polymer‐based nanoparticles, exosomes, and ligand‐RNA conjugates, represent safe and efficacious technologies and allow repeated administrations.^28^, ^29^ Moreover, lipid‐based nanoparticles have been the most extensively used for mRNA‐based therapeutics in preclinical studies and undergoing clinical trials (Table 1).^49^, ^50^ In addition, some nonviral vehicles possess targeting effects at desired sites in mRNA‐based therapeutics because of their organ‐targeted properties.^50^ Furthermore, nonviral nanoparticles are also substantially attractive in mRNA vaccines for virus outbreaks.^51^

**TABLE 1:** List of nanoparticle mRNA‐based vaccines in clinical trials

Nanocarrier	Disease	Names	General information	Clinical trials no./references
Cancer immunotherapy				
Liposomes (1,2‐di‐O‐octa decenyl‐3‐trimethyl‐ammonium‐propane (DOTAP), 1,2‐dioleoyl‐sn‐glycero‐3‐phosphoethanolamine (DSPC)	Melanoma	Lipo‐MERIT	Phase I, enrollment, completed, intravenously (IV) administered, elicit of T‐cell responses against malignant melanoma‐associated antigens	NCT02410733^30^
Liposomes	Triple‐negative breast cancer	TNBC‐MERIT	Phase I, active, not recruiting	NCT02316457^31^
Liposome	Ovarian cancer	W_ova1 vaccine	Phase I, active, not recruiting, IV injection	NCT04163094
LNPs	Colorectal cancer	RO7198457	Phase I, active, not recruiting, induction of both T‐lymphocyte‐ and memory T‐cell‐dependent immune responses against cancer ‐associated antigens	NCT04486378 NCT03815058
LNPs	Melanoma	Lipo‐MERIT	Phase I, active, not recruiting, IV injection, elicited T‐cell responses and tumor‐associated antigen	NCT02410733^32^
LNPs	KRAS mutant or metastatic cancer	mRNA‐5671/V941	Phase I/II, completed, intramuscular (IM) injection combination therapy	NCT03948763
LNPs	Metastatic melanoma, epithelial cancer, or gastrointestinal cancer	mRNA‐4650	Phase I/II, completed, IM injections, intramuscular safety, induced neoantigen‐specific T‐cell response	NCT03480152^32^
LNPs	Metastatic HPV16‐related squamous cell carcinoma of head and neck expressing PD‐L1	BNT113	Phase I, recruiting	NCT04534205^33^
LNPs	Melanoma Solid tumors	mRNA‐4157	Phase II, active, IM Injection, well tolerated	NCT03897881 NCT03313778 34
LNPs	Unmodified Melanoma	BNT111	Phase II, recruiting IV injection	NCT04526899 35
LNPs:	Relapsed lymphoma or malignancies	mRNA‐2416	Phase I, cessation, intratumoral injection	NCT03323398
LNPs	Solid tumor malignancies or lymphoma	mRNA‐2752	Phase I, recruiting, intratumoral injection	NCT03739931

Nanocarrier	Disease	Vaccination names	General Information	Clinical trials no./references
Prevent viral infection				
LNPs	Rabies virus	CV7202 (Rabies virus glycoprotein‐mRNA vaccine)	Phase I, completed, IM injection, neutralizing antibody responses; well tolerated	NCT03713086^36^
LNPs	Rabies virus	CV7201 (sequence‐modified mRNA of rabies virus glycoprotein)	Phase I, recruited, completed needle injection acceptable tolerability and generated rabies neutralizing antibody responses	NCT02241135 NCT03713086^36^, ^37^
LNPs	Human papilloma virus	HARE‐40	Phase I/II, recruiting, intradermal injection	NCT03418480^38^
LNPs	Human metapneumovirus and parainfluenza	mRNA‐1653 vaccine (nucleoside‐modified mRNA)	Phase I, completed IM injection	NCT03392389
LNPs	Cytomegalovirus Infection	mRNA‐1443, mRNA‐1647	Phase I, recruited, completed induce humoral and cellular immune response	NCT03382405 NCT04232280^39^
LNPs	Chikungunya virus	mRNA‐1388	Phase I, recruited, completed	NCT03325075
LNPs	Zika virus	mRNA‐1325, mRNA‐1893	Phase I/II, enrollment, completed well‐tolerated, induced a neutralizing antibody response	NCT03014089 NCT04064905
LNPs	Influenza viruses H10N8 and H7N9	mRNA‐1440 vaccine (nucleoside‐modified)	Phase I, enrollment, completed IM Injection reasonable safety, tolerability, stimulate humoral immune response	NCT03076385 40, 41
LNPs	Influenza H7N9	mRNA‐1851(H10N8 or H7N9 mRNA)	Phase I, completed IM injection well tolerability and immunogenicity	NCT03345043 40
LNPs	Respiratory syncytial virus (RSV), SARS‐CoV‐2	mRNA‐1345	Phase III, active, not recruiting IM injection induce protective immunogenicity	NCT05330975 NCT04528719
LNPs: ionizable lipid, cholesterol, DSPC, PEG2000‐DM	COVID‐19	mRNA vaccine (full‐length spike protein)	Phase I, completed IM injection induce neutralize antibody against SARS‐CoV‐2 infection	NCT04758962^42^
LNPs: ionizable amino lipid, PEGylated lipid, cholesterol, phospholipid	COVID‐19 SARS‐CoV‐2	mRNA vaccine (full‐length prefusion spike protein of SARS‐CoV‐2	Phase I, terminated IM injection induced significant humoral immune responses	NCT04860258^43^
LNPs: ALC‐0315, ALC‐0159, DSPC, cholesterol	COVID‐19 SARS‐CoV‐2	BNT162b2 (spike protein)	Phase III, completed, IM injection	NCT04816669^44^
LNPs: PEG2000‐lipid PEG2000‐DMG, DSPC, cholesterol	LUNAR‐COVID‐19	ARCT‐021	Phase II, terminated, IM injection	NCT04728347
LNPs: PEG‐lipid, cholesterol, 1,2‐dimyristoyl‐rac‐glycero‐3‐methoxypolyethylene glycol‐2000 (DMG‐PEG), DSPC, ionizable lipid	COVID‐19	mRNA vaccine (spike protein of SARS‐CoV‐2	Phase III, recruiting, approved by FDA, IM injection safety and well tolerability protection against SARS‐CoV‐2 infection	NCT04847102^45^
LNPs (ionizable lipid DSPC, cholesterol, DMG‐PEG)	SARS‐CoV‐2	mRNA‐1273 vaccine (spike protein mRNA vaccine) BNT162	Phase III, active, recruiting, approved by FDA, IM injection, 94.1% efficacy in protection against COVID‐19, feasible to storage and delivery	NCT04470427 NCT04537949 NCT04283461 NCT04860297^46^, ^47^, ^48^

Nanocarrier	Disease	Vaccination names	General information	Clinical trials no./references
mRNA for protein replacement				
LNPs	Ornithine transcarbamylase (OTC) deficiency	MRT5201 ARCT‐810 (human OTC protein)	Phase I/II, withdrawn, intravenous (IV) infusion, adequate enzyme replacement for correct OTC deficiency	NCT03767270 NCT04442347^2^
LNPs	Methylmalonic acidemia (methylmalonyl‐coenzyme A mutase (MUT) deficiency)	mRNA‐3927	Phase I/II, terminated, IV infusion	NCT03810690
LNPs (lipid, cholesterol, PEG2000, DSPC)	Propionic acidemia	mRNA 3927 (α and β subunits of mitochondrial propionyl‐CoA carboxylase protein)	Phase I/II, recruiting, IV infusion	NCT04159103
LNPs (Poly (β‐amino esters, PEG2000, DOPE, cholesterol)	Cystic fibrosis (CF)	MRT5005 (human CF transmembrane regulator protein)	Phase I/II, recruiting, aerosol inhalation	NCT03375047

Here, we reviewed the latest developments in mRNA‐based therapeutics, such as vaccination, genome editing, and protein replacement therapy. Moreover, we also emphasized the advanced delivery platforms for mRNA and their biomedical applications. In addition, we discussed the potential challenges of mRNA‐based therapeutics and provided our future perspectives.

## 2 mRNA FOR THERAPEUTIC APPLICATIONS

Over the past decades, mRNA‐based therapeutics have been highly promising for treating various human diseases, including cancers, infections, and autoimmune diseases.^52^, ^53^, ^54^ Recently, mRNA‐based vaccines have become a promising alternative in booster immunizations.^1^, ^55^ In addition, mRNA‐based protein replacement therapy provides a viable alternative and permanent cure for various diseases.^56^, ^57^ In 1961, as an intermediate hereditary substance, mRNA is first discovered.^58^ As early as 1990, scientists have first reported that intramuscular injection of in vitro‐transcribed mRNA into mouse skeletal muscle cells, results in in vivo expression of encoding proteins. Since then, mRNA‐based therapeutics have been exploited in a variety of application.^5^ In 2002, the first clinical trial used ex vivo mRNA to transfect dendritic cells (DCs), aiming to stimulate cytotoxic T lymphocyte against cancer.^59^ In 2020, mRNA‐based vaccines were approved against COVID‐19 by the U.S Food and Drug Administration (FDA).^60^

**FIGURE 1:**
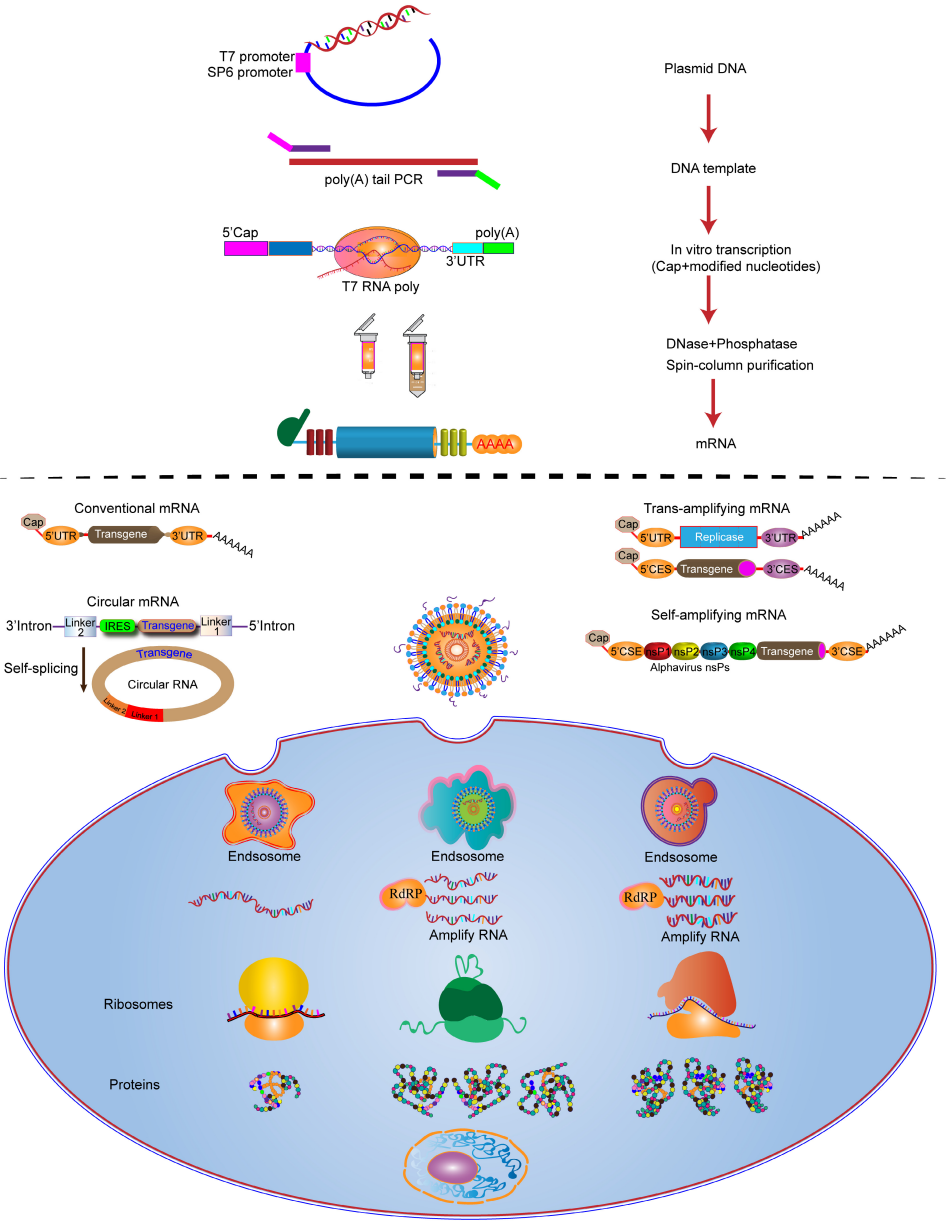
mRNA manufacturing process. In vitro transcribed mRNA encoding the gene of interest has been used to generate conventional mRNA. Use saRNA, which encodes a replicase that self‐amplifies mRNA for positive‐strand RNA viral genomes. The second utilizes a novel bipartite vector system of taRNA, in which the replicase is deleted to form a trans‐replicon. Both protocols improved the half‐life and translation efficiency of mRNA.

mRNA‐based therapeutics are composed of two main elements: the mRNA itself and the delivery systems. Most eukaryotic mRNAs have a tripartite structure comprised a 5′ cap structure, a 5′ untranslated region (5′ UTR), the open reading frame (ORF), a 3′ UTR, and a poly (A)‐tail to the 3′ end. The 5′ cap and 3′ poly a tail can protect mRNA from degradation by exonuclease, help bind to ribosomes, and initiate splicing, maturation, and translation.^61^, ^62^ The 5′ UTR and 3′ UTR are located at the upstream and downstream domains of the mRNA coding region, respectively, and are essential to protect the mRNA from decomposition and degradation.^63^ Moreover, the 5′ UTR plays a crucial role in controlling protein translation efficiency due to its binding site for the preinitiation complex initiation of protein translation.^64^ In addition, 3′ UTR contains degradation signals of mRNA, and alpha‐globin or beta‐globin 3′ UTR has been developed to improve the stability of heterologous mRNA in cells.^65^ The ORF region contains protein‐encoding nucleotide sequences with a start codon and ends with a stop codon.^66^ For the production of mRNA, the in vitro transcription (IVT) is performed on a linear pDNA template, and the RNA polymerase of T7 DNA48 synthesizes multiple copies of the RNA transcript.^67^ Once the RNA is capped at the 5′ end and purified, it is ready for formulation. Three main types of RNA are currently being developed: self‐amplifying RNA (saRNA), nonreplicating mRNA (nrRNA), and trans‐amplifying mRNA (taRNA)^68^, ^69^ (Figure 1). In this section, we summarized a detailed overview of mRNA‐based clinical applications, and mRNA deliver system is available in part 3.

### 2.1 mRNA‐based protein replacement therapy

Over the past few years, evidence has revealed that mRNA‐based therapeutics have gained immense interest and are demonstrating to be viable options in a wide range of diseases, including cancer,^70^ infectious disease,^71^ and rare genetic diseases.^72^ mRNAs can use the human body to translate and encode therapeutic proteins, such as transmembrane, intracellular, intramitochondrial, and secreted proteins.^73^ Therefore, mRNA‐based therapeutics can potentially restore the function of a missing or defective protein in diseased cells or create new cellular functions altering the diseased state.^74^

In mRNA‐based cancer immunotherapy, synthetic mRNA crosses the host cells through the cell membrane or by endocytosis and are translated to proteins in the cytosol. mRNA‐loaded DC vaccines can present major histocompatibility complexes (MHC) of the antigen‐presenting cells (APCs) to effector cells of the host immune system to induce cytotoxic T lymphocytes and natural killer (NK) cells attacking cancerous cells.^19^ Moreover, mRNA‐based anti‐tumor vaccines can help generate an immune‐friendly tumor microenvironment (TME) through activating toll‐like receptors (TLRs) and retinoic acid‐inducible gene I (RIG‐I) and secreting type I interferon, as well as chemokine GRO, MCP‐1, RANTES, and MDC.^75^, ^76^ In addition, mRNA can encode monoclonal antibodies (mAbs), playing a passive targeted immunotherapy strategy and achieving a more comprehensive oncology therapeutic effect.^77^, ^78^ Therefore, there are several ongoing clinical trials of mRNA immunotherapies for a variety of cancers.

Cardiovascular diseases (CVDs) lead to more than one‐third of all deaths worldwide, mainly due to ischemic heart disease (IHD).^79^, ^80^ Recent evidence has demonstrated that mRNA‐based management is promising to improve myocardial regeneration and prevent heart failure.^4^, ^81^, ^82^ The main mechanisms of IHD therapy include inhibition of cardiomyocyte death, promotion of cardiac regeneration, maintenance of coronary plaque, enhancement of angiogenesis, and triggering of cardiac reprogramming.^81^ For instance, pyruvate kinase muscle isoenzyme 2 (PMK2) is a critical enzyme in aerobic glycolysis, contributing to cell metabolic reprogramming.^83^ Furthermore, PMK2 can induce cardiomyocyte proliferation and is primarily expressed at higher levels in regenerative fetal heart and neonatal cardiomyocyte but not in adult cardiomyocyte.^84^ Therefore, in IHD therapeutics, the upregulation of PMK2‐encoding mRNA can re‐invigorate the proliferation of cardiomyocyte, resulting in cardiomyocyte regeneration.^84^ Moreover, it has been reported that insulin‐like growth factor‐1 (IGF1) protects cardiomyocytes after ischemia and myocardial infarction (MI).^85^ Therefore, promoting the expression of IGF1 in mouse hearts after MI can enhance cell survival and inhibit cell death under hypoxia‐induced apoptosis.^85^, ^86^ In addition, for MI treatment, overexpression of an angiogenic factor, human vascular endothelial growth factor A (VEGFA)‐encoding mRNA, in mice promotes the differentiation of heart progenitor cells into endothelial cells, improves heart function, and prolongs long‐term survival.^87^ Notably, mRNA‐based therapeutics play a vital role in injured hearts repair, angiogenesis, and cardiomyocyte regeneration.

mRNA‐based therapeutics have also become a new pillar for liver disease treatment. Crigle‐Najjar syndrome type 1 (CN1) is a rare metabolic disease characterized by the absence or decreased activity of UDP glucuronosyltransferase family 1 member A1 (UGT1A1), an enzyme required for glucuronidation of unconjugated bilirubin in the liver.^88^ The mutation of UGT1A1 gene contributes to severe unconjugated hyperbilirubinemia and consequently develops jaundice and irreversible brain and muscular damage.^89^ However, mRNA‐based therapeutics can restore the hepatic expression of UGT1A1 and normalize the levels of bilirubin.^90^ Moreover, α‐1 antitrypsin (AAT) deficiency is a recessive disease caused by mutations in the serpin family A member 1 (SERPINA1) gene.^91^ As a serine protease inhibitor, AAT neutralizes neutrophil plasminogen in the lung and liver to protect tissues from excessive proteinase activity.^92^, ^93^ The AAT deficiency can activate the intracellular injury cascade of apoptotic liver cell death, compensate hepatocellular proliferation, and lead to end‐organ injury.^94^ Nevertheless, supplement of AAT‐encoding mRNA can significantly produce AAT protein and provide an alternative modality for liver AAT deficiency.^95^ In addition, mRNA‐based therapeutics are currently being tested in preclinical and clinical studies for other liver diseases, including thrombotic thrombocytopenic purpura (TTP),^96^ glycogen storage disease type 1A (GSD1),^97^ acute intermittent porphyria,^98^ and factor IX deficiency hemophilia B.^99^ Therefore, mRNA‐based therapeutic are optimistic about liver disease treatment through the development of clinical studies.

Studies have shown that mRNA‐based therapy has outstanding advantages in treating neurological diseases. For instance, Kojima et al. have developed an emerging technique to delivery therapeutic catalase mRNA into the cytosol of nerve cell, which cannot only attenuate localized neuroinflammation but also rescue neuroinflammation in the model of Parkinson's disease. Moreover, mRNA‐based therapeutics also hold great potential for treating other diseases, such as leukemia,^74^ asthma,^100^ Fabry disease,^101^ skin disease,^102^, ^103^ and bone regeneration.^104^, ^105^

### 2.2 mRNA vaccines

**FIGURE 2:**
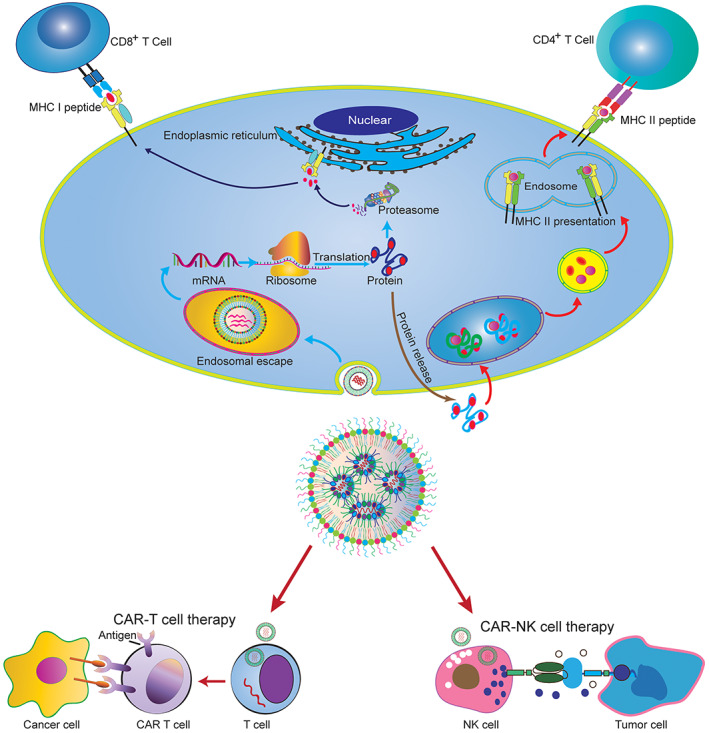
mRNA vaccines and mRNA delivery for CAR T‐cell/CAR NK‐cell engineering. Compared with other types of vaccines, mRNA vaccines have many advantages, including the use of noninfectious elements, reduced production time, and the potential to target multiple diseases. mRNA vaccines can be developed in the laboratory using DNA templates and readily available materials. Therefore, it can be standardized and mass‐produced, and the research speed of vaccines is faster than traditional vaccines. At present, COVID‐19 mRNA vaccines delivered by lipid‐derived nanoparticle (LNP) play a vital role in the fight against coronavirus as the first clinical mRNA vaccine.

Vaccines play a pivotal role in the public health and the quality of human life, which not only prevent various infectious diseases but also reduce and eradicate mostly fatal disease, such as *tuberculosis*, smallpox, ebola, meningitis, and malaria.^106^, ^107^ Traditionally, vaccines can be roughly subdivided into live attenuated vaccines, inactivated vaccines, subunit vaccines, and toxoid vaccines, which were commonly applied in clinical practice, lastingly provided protection for various diseases.^107^, ^108^, ^109^ Although conventional vaccines can provide long term and durable protection against diseases, they have difficulty in fulfilling pandemic needs as some emerging infectious diseases require more development speed, low‐cost manufacturing, and large‐scale production. mRNA vaccines have emerged as promising alternatives to traditional vaccines due to their advantages of rapid development, high efficacy, and cost‐efficient manufacturing. Furthermore, the mRNA vaccine is more likely to stimulate protective antibody and antigen‐specific T‐cell responses (Figure 2). Notably, the innate immune response is significantly enhanced following secondary immunization.^110^ In addition, conventional vaccines are not suitable for cancer vaccine development. In contrast, mRNA vaccines encoded using tumor‐associated antigens (TAAs) can be developed more rapidly with cost‐effective approaches.^111^, ^112^, ^113^

Moreover, mRNA vaccines have several advantages over conventional vaccines.^114^, ^115^ First, mRNA vaccines are scalable due to modified mRNA sequence can satisfy all genetic information requirements to encode all protein.^55^ Second, mRNA vaccines are comparatively effective and safe because they only target cytoplasmic delivery, avoiding the risk of genome integration.^116^ Third, manufacturing mRNA vaccines on a large scale tends to be industrialized.^116^ Therefore, mRNA vaccines have been widely studied in different kinds of diseases.

At present, there are several modes of production of RNA vaccines: template‐directed synthesis, nonreplicating, and self‐amplifying messenger RNA (samRNA) vaccines.^117^ Nonreplicating vaccines represent a straightforward approach wherein administered mRNA is directly translated into the cytoplasm of transfected cells to encode the protein antigens of interest.^118^ However, samRNA vaccines encode the RNA genome of a single‐stranded RNA virus and contain additional sequences in the coding region for RNA replication.^118^ However, both nonreplicating and samRNA vaccines have been proven effective against infectious diseases, and nonreplicating vaccines have strong potential to be used in cancer immunotherapy.^112^, ^115^, ^118^, ^119^ So far, most mRNA vaccines have been developed for infectious diseases or cancer immunotherapy. To our knowledge, most ongoing trials or FDA‐approved mRNA therapeutics are based on typical linear mRNA, which can be produced by RNA polymerase‐mediated IVT. However, in contrast to typical linear mRNA, circular mRNA (circRNA) increases mRNA stability and has been found to contribute to robust and durable protein expression.^120^ Therefore, the development of circRNA vaccines triggers higher and more durable effective neutralizing antibodies against severe acute respiratory syndrome coronavirus 2 (SARS‐CoV‐2) and shows adequate protection against new SARS‐CoV‐2 variants.^121^ In addition, these circRNA vaccines can strongly induce immune responses and are more durable than typical mRNA vaccines. However, the development of circRNA vaccines faces problems, such as efficient cyclization of intramolecular RNA, purification of circRNA, and high cost of IVT production reagents.^122^ We hope that advanced technology in circRNA by in vitro or in vivo synthesis can be developed in the coming years to facilitate the development of therapeutic circRNAs.

#### 2.2.1 mRNA vaccines for infectious diseases

mRNA vaccines against infectious diseases, such as influenza, Zika virus, and rabies, are underway in clinical trials. In contrast, the development is relatively slow due to mRNA stability and the lack of an optimized delivery system. However, the coronavirus disease‐2019 (COVID‐19) pandemic has accelerated the development and application of mRNA vaccines.^123^ COVID‐19 is a global pandemic caused by SARS‐CoV‐2, which has affected hundreds of millions of people and led to millions of deaths worldwide.^124^, ^125^ The rapid global spread of COVID‐19 has posed an international health emergency and economic burden as a devastating pandemic of the 21st Century.^126^

Two rapid‐response mRNA vaccines (mRNA‐1273 and BNT162b2) against COVID‐19 are licensed by the FDA, which are administered in the general population of many countries and have demonstrated excellent tolerability and high levels of protection against COVID‐19.^127^ Moreover, accumulating evidence has confirmed that the effectiveness of the BNT162b2 can achieve a protection rate of more than 90% against COVID‐19. In comparison, mRNA‐1273 has a protection rate of 87.4%, decreasing hospitalization and hospital mortality by 95.8% and 97.9%, respectively.^128^, ^129^ Unfortunately, the effectiveness and protection of these vaccines are reduced due to SARS‐CoV‐2 variants, including alpha (B.1.1.7 and descendant lineages), beta (B.1.351), gamma (P.1), delta (B.1.617.2 and AY lineages), and omicron (B.1.1.529 and BA lineages).^130^ Therefore, further improvement and development of mRNA vaccines are necessary to protect the constantly evolving SARS‐CoV‐2 virus. Fortunately, multiple mRNA vaccines have been designed against acute infectious viruses, such as flaviviruses, influenza viruses, and rabies virus, and chronic infectious viruses, such as hepatitis C virus (HCV) and human immunodeficiency virus (HIV).^131^ Moreover, most of these mRNA vaccines have been in the phase of clinical trials.^131^

#### 2.2.2 mRNA vaccine for cancer therapy

The clinical application of mRNA‐based tumor vaccine has achieved significant progress over the past decade.^119^, ^132^ mRNA‐based vaccine is an innovative administration of cancer immunotherapy, and the vaccine plays a vital role in delivering the antigens recognizing by the body's immune cells to induce immune response.^113^, ^133^ Moreover, mRNA‐based vaccines can code tumor‐specific antigens (TSAs), introduce them into APCs, and synthesize the required antigens, stimulating both humoral and cellular immunity to kill cancer cells.^115^ In addition, mRNA‐based vaccines can also encode and express TAAs and TSAs to specifically attack and eliminate cancer cells.^134^

To date, some strategies have demonstrated that mRNA‐based vaccines possess the feasibility and tremendous potential to be applied in cancer treatment. For instance, BI1361849, as an mRNA encoding non‐small cell‐associated tumor antigens, can promote the proliferation of functional tumor‐specific CD4 and CD8 cell, killing the cancer cell and improving survival.^135^ In addition, DCs‐based mRNA vaccines also provide the necessary modalities to administer the tumor. DCs, as the most efficient APCs, play a vital role in the activation process of helper T and killer T cells through capturing, processing, and presenting antigens to T cells.^136^ The DCs‐based mRNA tumor vaccines are produced undergoing multiple procedures: DCs are extracted from the patient's peripheral blood, and the differentiation and maturation are stimulated using the granulocyte‐macrophage colony‐stimulating factor (GM‐CSF) and interleukin‐4 (IL‐4); then mRNA encoding TSAs is transfected; the mRNA‐loaded DCs are finally reinfused into the patients, and the immune system is stimulated to combat cancer cells.^137^ For example, the pp65‐mRNA‐loaded DCs vaccine is used to treat glioblastoma, and it can increase long‐term progression‐free survival and prolong overall survival.^138^ All these studies show that mRNA‐based vaccines may be safe and effective alternatives to cancer immunotherapy. In addition, many mRNA‐based cancer vaccines have been evaluated for their effectiveness.

### 2.3 Genome editing by mRNA

In recent years, genome editing has become the most potent strategy in the field of gene therapy. Zinc finger nuclease (ZFN), transcriptional activator‐like effect nuclease (TALEN), and clustered regularly interspaced short palindromic repeat sequence (CRISPR)‐related protein 9 (Cas9) systems have been developed as powerful tools for locus‐specific modification of genomes, providing a promising strategy for diseases caused by gene defects.^139^, ^140^ However, these approaches have disadvantages of the risk of off‐target mutagenesis.^141^ Consequently, mRNA‐based encoding ZFNs, TALENs, and CRISPR/Cas9 has been successfully applied for genome editing in vivo and in vitro, providing an attractive alternative due to their transient expression.^141^

**FIGURE 3:**
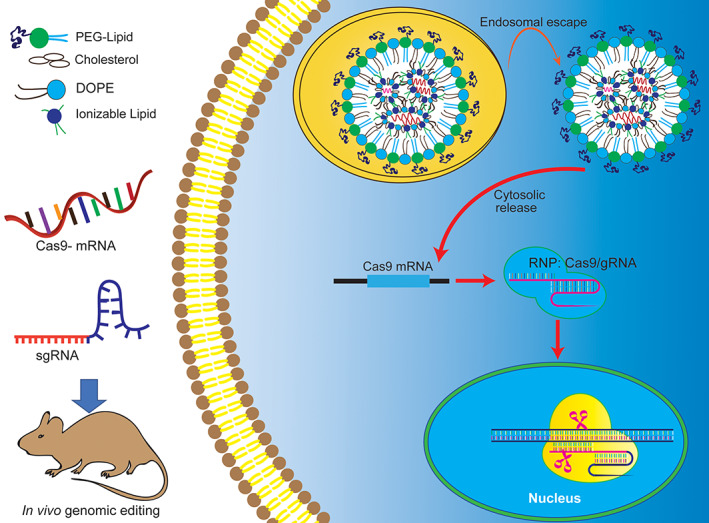
Delivery of mRNA for genomic editing by nanoparticles. The CRISPR/Cas9 components (sgRNA, Cas9 mRNA) are encapsulated in nanocarriers, which need to escape from endocytosis/lysosomes after cellular uptake. Then, it will be translated directly into proteins in the cytoplasm and transported to the nucleus so that the CRISPR machinery can act on the genomic DNA of the cell. mRNA delivery and translation are transient and thus avoid the concern of permanent integration of CRISPR genes into the host genome.

mRNA‐based genome editing open the opportunity to be used in various therapeutic field as a novel drug candidate. For example, ZFNs‐encoding mRNA is used to target the human C‐C chemokine receptor 5 (CCR5) for the treatment of HIV.^49^, ^142^ Moreover, TALEN‐encoding mRNA treats pyruvate kinase deficiency (PKD) by correcting the pyruvate kinase L/R gene (PKLR) in hematopoietic stem cells.^143^ In addition, nanoparticle‐mediated CRISPR/Cas9‐encoding mRNA delivery is the most frequently used gene‐editing technology (Figure 3). Several studies have shown that in non‐human primate models, CRISPR/Cas9‐mediated CCR5 disruption is used to give resistance to HIV/SIV infection.^144^ Therefore, mRNA‐based genome editing will be a promising modality for gene therapy, and several of them are undergoing clinical studies.

## 3 NANOPARTICLES FOR MRNA DELIVERY

mRNA must to enter the host cytoplasm and express specific antigens to remain functional. However, it is difficult for negatively charged mRNA macromolecules to penetrate the anionic lipid bilayer on the cell membrane. Moreover, the single‐chain mRNA is fragile and easily degraded by extracellular ribonucleases in skin and blood.^9^ Therefore, an ideal mRNA delivery system not only enhances efficient cellular uptake by host cells but also protects them from degradation. In the following sections, we describe various mRNA delivery systems developed and applied.

### 3.1 Lipid and LNPs for mRNA delivery

**FIGURE 4:**
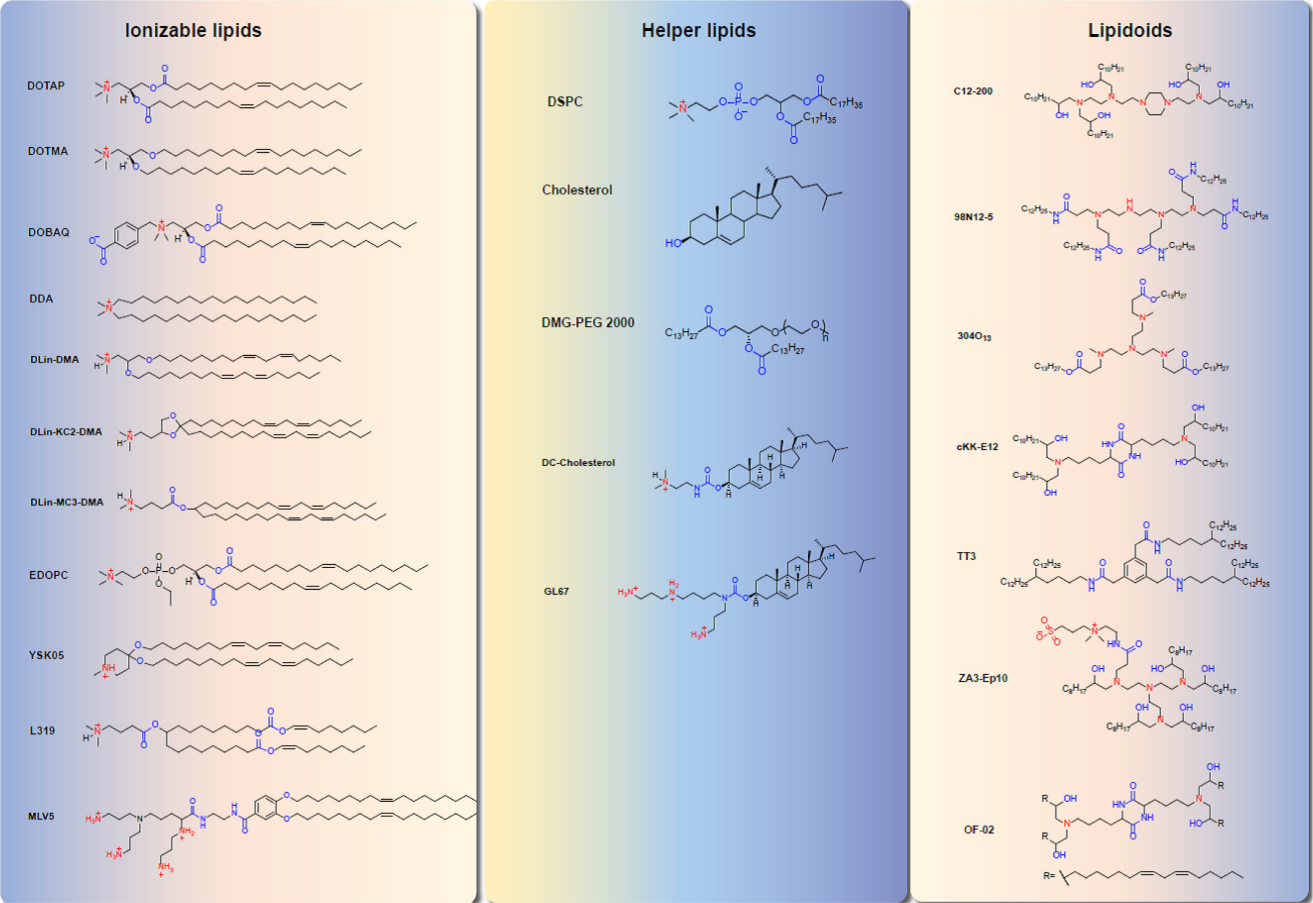
The chemical structural formula of major lipids utilized by lipid nanoparticles for mRNA‐Based therapy.

The lipid can be cationic, neutral, and zwitterionic, which is an important class of vector material for mRNA delivery.^145^ However, cationic lipids represent the widely applied and studied materials for mRNA delivery.^145^ For example, N‐[1‐(2,3‐dioleyloxy) propyl]‐N,N,N‐trimethy‐lammonium chloride (DOTMA), as the first synthetic cationic lipids, is used to delivery mRNA to several cell lines.^146^ Unfortunately, toxicity and immunogenicity may be the main obstacles to its clinical application.^147^ The cationic lipids, combined with other components forming nanoparticles, are named LNPs.^148^ LNPs are the most extensively investigated and clinically advanced delivery system for mRNA‐based therapy.^13^, ^149^ LNPs are nanocarriers with a monolayer structure of phospholipids, which can protect the mRNA in the lipid core from degradation. Usually, LNPs are synthesized by cholesterol, polyethylene glycol‐modified (PEGylated) lipids, helper phospholipids, and ionizable lipids.^13^ Figure 4 represents the structural classes of lipids components of LNPs tested for mRNA‐based vaccines and treatments. In addition, each component of LNPs exerts a different function in mRNA delivery. Cholesterol can enhance the stability of lipid nanoparticles, assist membrane fusion, and facilitate the entry of mRNA into cytoplasm.^150^ The helper phospholipids are generally saturated phospholipids, which can improve the overall phase transition temperature and stability of nanoparticles.^151^ PEGylated lipids can potentially improve the manufacturability and stability of LNPs and prevent macrophage‐mediated degradation.^152^ Ionizable lipids are the most critical excipient and decisive factor in releasing mRNAs in the cytoplasm through ionization.^153^, ^154^ Therefore, LNPs have been widely utilized as the vehicle for mRNA drugs. For instance, LNPs‐mediated delivery of IL‐10 mRNA can promote the expression of IL‐10 in inflammatory leukocytes and attenuate the dextran sodium sulfate (DSS)‐induced colitis in experimental mouse.^155^ Moreover, LNPs mediated delivery of mRNA vaccines against COVID‐19 has confirmed encouraging results and shown a perfect protective efficacy across different population.^60^, ^127^ In addition, LNPs mediated delivery of Cas9 mRNA is to exert cancer immunotherapy or genome editing.^156^, ^157^, ^158^

Despite their unprecedented potential, several studies have demonstrated that LNPs still have potential limitations that hinder them practical application. One limitation is the safety of LNPs due to their potential toxicological effects and the tissue distribution of their payloads.^159^, ^160^ Another limitation is immunogenicity of LNPs because LNPs constituents can induce immune response, promote neutrophil infiltration, and boost the secretion of proinflammatory factor and reactive oxygen species (ROS).^161^ Moreover, LNPs induced side effects, such as pain, redness, and fever, have been reported.^162^, ^163^ Therefore, further studies on the cytotoxicity and immunogenicity of LNPs delivery system are urgently needed.

### 3.2 Lipopolyplex for mRNA delivery

The lipopolyplex (LPP) nano‐delivery platform is a bilayer structure with a polymer‐encapsulated mRNA molecule as the core, which is wrapped in a phospholipid‐coated bilayer shell to protect the mRNA molecule in the polyplex core structure from RNase degradation. As a nonviral gene delivery vector, LPP combines the advantages of polymers and liposomes, exhibiting superior stability, reduced cytotoxicity, high gene transfection efficiency, and the ability to release mRNA molecules progressively with polymer degradation. For example, Persano et al. have developed a lipopolysaccharide (LPS) mRNA vaccine consisting of a poly(β‐amino acid) polymeric mRNA core wrapped inside a lipid shell. Intraperitoneal injection of LPP carrying ovalbumin antigen and tyrosinase‐related protein 2 (TRP2) mRNA can target DCs and activated T‐cell immune responses and melanoma lung metastases in a mouse model of the tumor, with over 90% reduction in tumor nodules nodule size were observed.^164^ In contrast to liposome delivery of mRNA, LNP delivery of N1 methyl pseudouridine‐modified mRNA cannot induce type I IFN for effective T‐cell immunity. This mode of action can reduce the inflammatory response without impeding T‐cell immunity. This approach provides a new way to explore effective and low inflammatory mRNA lipid polymorphs.^165^

### 3.3 RNA delivery with virus‐like nanoparticle

Virus‐like nanoparticle (VLP) delivery system is an intermediate between viral and nonviral vectors. These particles contain most components from viral vector, such as envelope and capsid but no viral genome.^166^ Specific recognition between mRNA stem‐loop structure and viral capsid structure capsid protein can provide VLP‐mRNA formation by virus engineering technology. In particularly, VLP‐mRNA can efficiently infect cells with the help of viral capsid and transient expression of mRNA itself. It has been shown that Cas9 mRNA delivered by VLP‐mRNA can be present for only 72 h compared to viral systems expressing Cas9 for a long time. Therefore, the off‐target effects can be significantly reduced. VLP delivery of cas9 mRNA has successfully developed Vegfa and reduced neovascularization area by 63% as a significant therapeutic approach in a mouse model of age‐related macular degeneration. Sequence results show that VLP‐mRNA does not induce off‐target effects.^167^ These experimental results strongly support the potential of VLP for clinical application in delivering CRISPR gene therapy.^168^ Yadav et al. have fused an aptamer‐binding protein (ABP) to the N‐terminal of the HIV Gag protein. The interaction between RNA aptamers and ABPs can recruit Cas9 mRNA or RNP for enhanced vector production and gene expression.^169^ Segel et al. have found that one of the retroviral‐like proteins (PEG10) directly binds to an extracellular viral‐like capsid and secretes its mRNA into the vesicle. The mRNA cargo using PEG10 can be engineered to package, secrete, and deliver specific RNAs by linking the target mRNA to the UTR of PEG10, and PEG10‐based VLP have low immunogenicity compared to currently available viral vectors, dramatically expanding the range of applications for nucleic acid therapy.^170^ As a naturally occurring biodegradable nanomaterial, VLP avoids disrupting the genome integrity raised by retroviruses. With the further development of gene technology, VLPs are being developed as safe and effective vaccines and gene editing delivery tools.

### 3.4 Membrane‐coated nanoparticles/biomimetic nanoparticles

Cell membrane‐coated vehicles can mimic the properties of cell membranes, which combine the properties of natural cell membranes with those of nanomaterials, thus significantly improving biocompatibility while enabling long circulation and targeted delivery in vivo. A series of nano‐drug carriers have been developed using immune cell membranes, such as leukocytes, macrophages, neutrophils, and other “camouflage.” In addition, tumor cells and bacteria can be used to prepare cell membrane‐camouflaged nanocarriers. Given the specific proteins on the surface of tumor cells and bacteria that activate the immune system and improve adhesion, these new drug carriers are more diverse in function delivery of mRNA drug than traditional nanocarriers. Moreover, the utilization of specific recognition proteins on cell membranes can achieve targeted drug delivery to lesion sites, offering a novel strategy for significantly improving the therapeutic effect. Despite the apparent advantages of membrane‐coated nanoparticles (CM‐NPs), technical preparation for such CM‐NPs remains challenging.^171^ Therefore, much work remains to be explored before their clinical implementation.

Meanwhile, virus‐mimicking cell CM‐NPs promise to improve cellular delivery efficiency. Previous studies have shown that the hemagglutinin (HA) protein or HA2 peptide derived from the surface of the influenza virus mediates membrane fusion in the endosomal acidic pH environment.^172^ Therefore, researchers genetically modified cells to express the HA protein or insert HA2 peptide on the cell membrane to create such nanoparticles. They have then isolated cell membranes coated mRNA/miRNA nanoparticles inside.^173^ This virus‐mimicking cell MC‐NP can trigger the endosomal escape and release its mRNA/miRNA load to reach intracellular localization for protein regulation.^174^

### 3.5 Polymer‐based delivery system

The polymer‐based delivery system consists of another prominent family of mRNA delivery carrier and has excellent potential for mRNA delivery.^175^ Moreover, a polymer‐based delivery system is comprised three kinds of polymer, including cationic polymer, dendrimer, and polysaccharide polymer.^13^ Cationic polymers have the advantages of easy synthesis and modification. In addition, cationic polymer can be complex with mRNA by electrostatic attraction and hydrophobic interaction, contributing to a more stable polyplex.^176^ Polyethyleneimine (PEI) is one of the most widely used network polymers and has high efficiency of mRNA delivery.^177^ However, PEI has the disadvantages of poor biodegradability and high toxicity, limiting its broad clinical application.^147^ Fortunately, with the development of chemical modification, the toxicity of PEI is reduced while maintaining its protonatable properties and high delivery efficiency.^178^ For example, Li et al. have found that linking cyclodextrin to PEI not only reduces the cytotoxicity of cationic PEI polymer but also maintains a large number of protonatable groups. Moreover, they have further discovered that modified PEI‐based mRNA encoding HIV‐1 gp120 can be dramatically up‐taken by lymphoid tissue, resulting in anti‐HIV immune response in experimental mice.^178^

Dendrimer polymer is a vital delivery vehicle with high tolerability, which contains a core and multiple regularly hyperbranched monomers.^179^, ^180^ Results from Xiong et al. show that the delivery system based on dendrimer polymer can successfully transport many kinds of mRNAs to tumor tissue, which provides convenient for tumor radiography and treatment.^181^ Moreover, the etiology of hepatorenal tyrosinemia type I (HT‐1) is involved in the mutation of fumarylacetoacetate hydrolase (FAH), which prevents catalysis of the terminal step in the tyrosine catabolic pathway, resulting in an aggregation of toxic metabolites and the disfunction of liver and kidney.^182^ Interestingly, the delivery of therapeutic FAH mRNA based on dendritic molecules can restore liver function and prolong the survival time of HT‐1 mice.^183^ Therefore, these results provide a dendrimer polymer delivery system with great potential to improve mRNA delivery and treat genetic diseases.

Polysaccharides, as relatively common natural biomaterials, can be easily chemically modified for efficient delivery with high biocompatibility and little toxicity and are mainly composed of chitosan, alginate, dextran, and hyaluronic acid.^184^ Chitosan is one of the most common polysaccharides to deliver mRNA. It has been reported that chitosan‐based deliver therapeutic surfactant protein B (SP‐B) mRNA can replace SP‐B deficiency and experimental asthma in mouse models and prolonged life in treated mice.^185^ Cystic fibrosis (Cf) is a genetic disorder that originates in an alteration in the cystic fibrosis transmembrane conduction regulator (CFTR) gene.^186^, ^187^ Fortunately, chitosan‐based delivery of CFTR mRNA can restore CFTR function and provide great potential for treating CF.^188^ Alginate, as an important polysaccharide, is derived from brown algae and bacteria and consists of a‐l‐guluronic acid and b‐d‐mannuronic acid building blocks linearly linked by 1,4‐glycosidic linkages.^189^ Moreover, alginate has biodegradable, nontoxic, and abundant features.^190^ It has been reported that delivery of alginate‐based neurotrophin‐3 (NT‐3) mRNA can enhance nerve regeneration.^191^ Dextran, as a water‐soluble, naturally degradable polysaccharide, originates from bacterial metabolites and consists of (1,6)‐a‐d‐glucose with various ratios of linkages and branches.^190^ It has been demonstrated that dextran‐based mRNA delivery can induce high gene expression and low cytotoxicity and provide a promising strategy to treat breast cancer.^192^ Hyaluronic acid, as a nonsulfated glycosaminoglycan, is composed of glucuronic acid and *N*‐acetyl‐d‐glucosamine bound linked beta‐linkages with good bio‐compatibility and biodegradability.^193^ Yan et al. have found that hyaluronic acid‐based Wingless and the name Int‐1 (WNT) 16 mRNA can be delivered to human cartilage explants and antagonize canonical β‐catenin/WNT3a signaling, contributing to increased lubricin production and decreased chondrocyte apoptosis in osteoarthritis (OA).^194^ Therefore, polysaccharides polymer may be a promising substitute to deliver mRNA in treating human diseases.

### 3.6 Inorganic‐based nanomaterial for mRNA delivery

Inorganic nanomaterials as delivery vehicles have unique physicochemical properties, such as excellent storage stability, good biocompatibility, and easy preparation, making them ideal platforms for mRNA delivery. Currently, inorganic nanostructures, including quantum dots, silica nanoparticles (SNPs), gold nanoparticles (GNPs), and carbon‐based and magnetic iron oxide‐based nanostructures, are the most popular types in the field of nanomedicine. AuNPs exhibit unique properties for delivering nucleic acid drugs. Through covalent or noncovalent conjugation, functional moieties, such as nucleic acid drugs and targeting ligands, can covalently attached to the gold nanoparticle core through a thiol moiety. AuNPs are expected to be attractive scaffolds for mRNA delivery due to their tunable size, simple functionalization, nontoxicity and immunologically inert properties. Yeom et al. have observed that mRNA encoding the pro‐apoptotic factor Bcl‐2‐associated X (BAX) protein is encapsulated by AuNPs, showing highly efficient mRNA delivery and inhibiting xenograft tumors.^195^ The ligand length, density, particle size, hydrophobicity, and affinity of AuNPs need to be considered and optimized for efficient, targeted delivery of mRNA nanoparticles. Iron oxide nanoparticles are nanoscale magnetic materials mainly composed of iron‐based oxides, which have been widely used in biomedical research due to their superparamagnetic properties at specific sizes, biocompatibility, and easy‐to‐modify surfaces for biologics. For example, iron oxide nanoparticles coated with nucleic acid‐loaded lipidoids exhibit efficient RNA and DNA delivery in vitro. Most recently, Erasmus JH et al. developed a lipid in organic nanoparticle (LION) consisting of inorganic superparamagnetic iron oxide (SPIO) nanoparticles for delivery saRNA, which is undergoing clinical trials for the protection against SARS‐CoV‐2 infection.^196^ This formulation is highly stable for at least 3 months when stored between 4 and 25°C. The showcases the role of SPIO nanoparticles in enhancing the stability, delivery, and immunogenicity of mRNA vaccines. However, the long‐term, nonspecific accumulation of these inorganic nanomaterials in organisms may cause toxicity, which hinders their large‐scale clinical applications. Detailed toxicological assessments (genotoxicity and oxidative stress) are therefore needed. It is crucial to develop biodegradable and scavengable inorganic nanomaterials to minimize the short‐ and long‐term cytotoxicity of inorganic nanoparticles. Besides, these inorganic nanomaterial carriers must be targeted to specific tissues or organs to minimize side effects.

### 3.7 Exosome‐based nanoparticles for mRNA delivery

**FIGURE 5:**
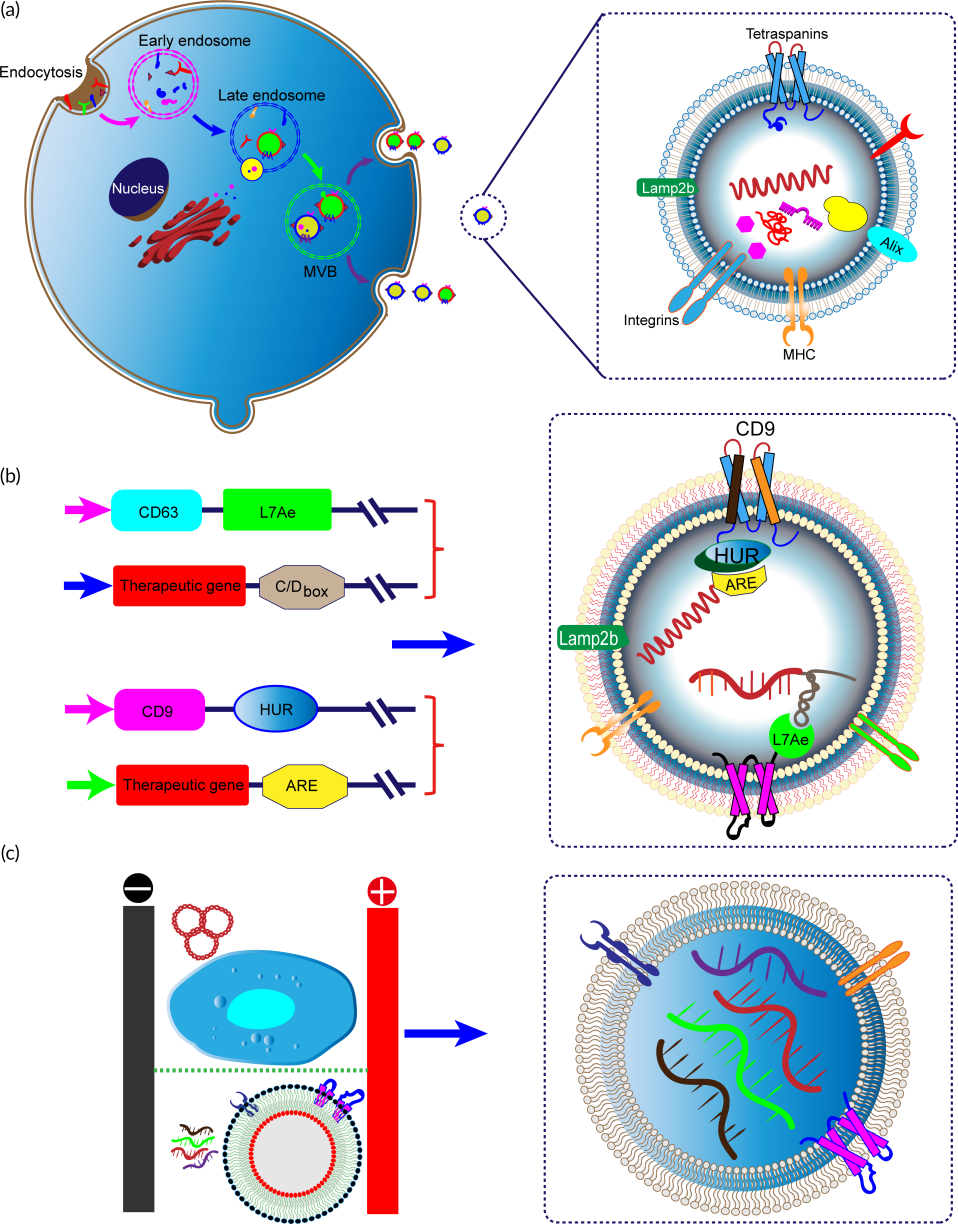
Exosome‐mediated mRNA delivery for gene therapy. (a) Exosomes are biogenesis from the invagination of the cell membrane and mature into late endosomes/MVBs. Then, MVBs fuse with the cellular membrane and are released into the extracellular space. (b) Packaging and delivery of RNAs via binding with RNA‐binding protein (HUR or L7Ae) that recognizes specific sequences on RNA. (c) Cellular‐nanoporation technology for large‐scale generation of exosomes containing therapeutic mRNAs

Exosomes are engendered by the inward budding of the endosomal membrane forming Intraluminal vesicles (ILVs) within multivesicular bodies (MVBs). When MVBs fuse with the plasma membrane, the ILVs are released into the periplasmic space referred to as exosomes (Figure 5). As a group of small vesicles (50–150 nm), exosomes contain large amounts of specific proteins, lipids, DNA, mRNA, noncoding microRNAs (microRNAs and miRNAs), and enzymes, which are a new mode of intercellular communication. Therefore, exosomes can function as an mRNA cargo delivery system.^197^ Table 2 summarizes the recent progress of exosome‐based mRNA delivery systems for treating various diseases. In contrast to synthesized nanoparticles, native exosomes possess outstanding properties, such as excellent biocompatibility and low immunogenicity. In addition, due to the small size of exosomes, it can inhibit the clearance by mononuclear phagocytes and has high permeability and retention effects at solid tumor sites, which can achieve drug accumulation at target sites. Exosomes themselves can hijack the blood–brain barrier (BBB) to deliver drugs into the brain. Especially, intranasal administration can promote the drug to reach the brain lesions quickly, providing the possibility for noninvasive treatment of brain diseases. Using the advantages of exosomes across the BBB, Kojima et al. have encapsulated catalase mRNA in HEK293T‐derived exosome to effectively attenuate neuroinflammation in the model of Parkinson's disease or LPS‐induced rats.^203^ Yang et al. have employed exosomes to encapsulate phosphatase and tensin homolog (PTEN) as tumor suppressor for brain cancer treatment.^202^ In this way, gliomas‐targeting peptides are *anchored* to the N‐terminus of exosomal membrane protein CD47, which remarkably enhances the cellular uptake of exosomes by glioma cells. These modified glioma‐targeted exosomes exhibit a significant inhibitory effect on tumor cell dissemination without any side effects on other normal tissues.

**TABLE 2:** Recent advances in exosome‐mediated mRNA therapy and genomic edit

Donor cells	Cargo	Loading method	Function	References
HEK293T RAW264.7	Interleukin‐10 mRNA	Cells were transfected with IRES‐IL‐10 expressing vector plasmids	Reduces atherosclerotic lesion formation	198
AML12 cells	Ldlr mRNA	Cells were transfected with Ldlr plasmids	Ameliorate the phenotype of atherosclerosis	199
Bone marrow‐derived MSCs	LDLR mRNA	Ldlr‐expressing virus vector	Homozygous familial hypercholesterolemia patients	Clinical trials: NCT05043181
HEK293	Nerve growth factor (NGF) mRNA	Cells were transfected with NGF plasmid	Reduce inflammation and rescue ischemic injury	200
HEK‐293 T	UPRT mRNA	Transfected cell with pCD‐UPRT‐EGFP plasmid	Activate pro‐drug and inhibit schwannoma tumor growth	201
MEFs, MSCs, DCs or HEK‐293 T cells	PTEN mRNA	Cellular nanoporation	Restore tumor‐suppressor function, produce larger amount of PTEN mRNAs into exosome	202
HEK‐293 T (EXOtic devices)	Catalase mRNA	Cell were transfected plasmids encoding catalase mRNA (PhCMV‐Catalase‐C/Dbox‐pA)	Protection against neurotoxicity and neuroinflammation	203
293FT	HCHrR6 mRNA	Transfection of cells with the XPort/HChrR6 encoding plasmid	Targeted delivery and inhibit tumor growth in vivo	204
293F	SARS‐CoV‐2 spike and nucleocapsid proteins	Incubation with lipid‐coated mRNAs	Induce long‐lasting cellular and humoral responses	205
Red blood cell‐derived EVs (RBCEVs)	Cas9 mRNA	Electroporation	Improve genome editing	206
SK‐LU‐1 Lung adenocarcinoma cells	p53 mRNA	Transfected with wt‐p53 expressing plasmid DNA	Support antitumor environment leading to decreased oncogenesis	207
HEK293T cells	CRISPR/dCas9 mRNA	CD9‐HuR and AU rich elements	Effectively deliver CRISPR/Cas9 system and reduced C/ebpα expression in the liver	208
HEK293T cells	GFP mRNA p53 mRNA	Co‐transfected with ARRDC1‐TAT and TAR‐GFP/TAR‐p53	Efficiently package and deliver mRNA	209
HEK 293 T cells human lung spheroid cells	Spike‐mRNA GFP‐mRNA RFP‐mRNA	Electroporation	Dry powder available drug‐delivery vehicles inhalation of mRNA	210

Mizrak et al. have exploited the genetically engineered microvesicles (MVs) derived from parent cell overexpressing suicidal CD‐UPRT mRNA. They have found that MVs deliver CD‐UPRT mRNA into schwannomas via direct intratumoral administration, which can achieve high‐level expression of functional proteins and promote tumor cell regression following systemic treatment with the prodrug 5‐FC.^203^ Exosomes can package synthetic mRNA from overexpression of RNA in parental cells by DNA transfection or lentiviral transduction. Therefore, mRNA can be passively loaded into exosomes by transient transfection of IL‐10 in parent cell. Exosome‐based engineered IL‐10 mRNA offers an efficient drug delivery vector target to macrophages in plaques of experimental atherosclerosis and alleviates atherosclerosis in ApoE^−/−^ mice. Therefore, it provides a safe and efficient approach to treat atherosclerosis.^198^ According to a recent study by Wang et al., exosomes were used as carriers to improve the delivery of mRNA encoding HChrR6 to target cells (HER2+ cells).^204^ Li et al. have demonstrated that exosomes from Ldlr overexpressing liver cells can protect against atherosclerosis. For the applicable clinical trial, they have used GMP‐compliant normal donor bone marrow‐derived MSCs to yield Ldlr mRNA‐enriched exosomes. They plan to inject exosomes via ultrasound‐guided paracentesis as a first‐in‐human study to evaluate the safety of this exosome product for gene therapy. This study is expected to provide a new therapeutic strategy for patients with homozygous familial hypercholesterolemia (Clinical Trials: NCT05043181).

Exosomes have received increasing attention as promising vehicles for the delivery of mRNA vaccines, especially those against tumors and Covid‐19, as DC‐derived extracellular vesicles containing MHCs on their surface can enhance T‐cell immune responses in patient.^211^ In the further development of exosome as vaccines, the delivery of mRNA of pathogenic proteins into DC‐derived may provide a rich source of antigens.^211^ In this approach, total tumor RNA is loaded onto DC‐derived extracellular vesicles. Exosome‐mediated mRNA delivery is safe and has high clinical potential for inducing SARS‐CoV‐2 immune function. Compared with LNPs that cause significant cytotoxicity, exosomes have no side effects in vitro or in vivo at any dose tested. Furthermore, exosomes medicate functional mRNA to human cells showing better performance than mRNA‐loaded LNPs. Exosomes incorporated with mRNA encoding spike protein of SARS‐CoV‐2 can significantly induce long‐term immune responses.^205^ Collectively, these results reveal that exosomes can be utilized as deliver carrier for mRNA against COVID‐19.

Furthermore, traditional transfection methods can directly load the purified exosomes with synthetic mRNA molecules. After the electroporation, small micropores appear on the surface of exosomes, and the mRNA can enter the vesicle along the transient small hole. Electroporation has been widely used as a technology for loading exogenous mRNA. For example, extracellular vesicles from human erythrocytes have also been electroporated with mRNA‐encoded Cas9 and gRNA targeting MiR‐125b‐2 locus, showing genome editing in leukemia cells^206^ (Figure 5a). However, the mRNA of the gene‐editing protein Cas9, with a size of nearly 5000 nt, makes it challenging to encapsulate CRISPR/Cas9 into exosomes by electroporation or other strategies. For specific enrichment of CRISPR/Cas9 mRNAs into extracellular vesicles, the exosomal transmembrane protein CD9 is fused with HuR protein, which can facilitate the sorting of RNAs with AU‐rich elements into exosomes. Li et al. have shown that Cas9 mRNA engineered with AREs (AU‐rich elements) can be efficiently loaded into exosomes and significantly reduce the expression of C/ebpα in the liver.^208^, ^212^, ^213^ Previous evidence has suggested that the ribosomal protein L7Ae forms a classic kink‐turn structure with the C/D box RNA structure,^214^ and the resulting protein–RNA complex can serve as a unique way to load mRNA (Figure 5b). Kojima et al. have assembled L7Ae before the N‐terminus of CD63 and fuse the C/D box at the end of the 3′‐UTR of the target gene. Interactions between RNA‐binding protein and the box C/D motif can specifically recruit RNAs into exosome.^203^ Another approach to precisely manipulate exosomes utilizes a specific subset of MVs termed “arrestin domain‐containing protein 1 (ARRDC1)‐mediated MVs (ARMM).” In this method, the C‐terminus of ARRDC1 is fused to a TAT peptide that mainly reacts with TAR and a stem‐loop‐containing transactivation response (TAR) element. RNA cargo molecule can be efficiently loaded into ARMM. GFP and p53 mRNAs are successfully delivered into exosomes by this strategy and expressed in recipient cells.^209^ To improve the mRNA‐loading efficiency of exosomes, James Lee's group have reported a cellular nanoporation method to produce large amounts of therapeutic mRNA‐containing exosomes^202^ (Figure 5c). The production of RNA‐loaded exosomes can be further promoted by mechanically extruding the vesicles through a micrometer pore filter using cells expressing of the desired mRNA.^215^

In addition to exosomes as emerging carriers for mRNA drug delivery, surface functionalization of exosomes can achieve target‐specific delivery.^216^ For example, we used targeted peptide expressed with Lamp2b on the external surface of exosomes to target chondrocytes or mesenchymal stem cells.^217^, ^218^, ^219^ Thereby, modified exosome can precisely delivery mRNA molecules to target cells or organs. Overall, exosomes are highly biocompatible and have substantial clinical application potential, opening up a new avenue for mRNA drug delivery.

## 4 CONCLUSION AND FUTURE PERSPECTIVES

The rapid development of mRNA therapy has brought unprecedented prospects and opportunities in the biomedical field. We comprehensively reviewed the latest progress in mRNA delivery, which gives essential value for designing mRNA delivery systems. Addressing the poor stability of mRNA for in vitro and in vivo target delivery is a crucial aspect of its clinical application. Meanwhile, mRNA has a large molecular weight and is negatively charged, making it challenging for exogenous mRNA to enter the cells. However, clinical trials have shown that physical methods are often harmful to cells and are unsuitable for in vivo application. Although viral vector‐based delivery of mRNA is highly efficient and usually shows better transfection efficiency, the undesirable properties of viral vectors, including potential carcinogenicity and high immunogenicity, hinder its broad clinical application. Until the outbreak of the COVID‐19 pandemic in 2020, mRNA‐based therapy was used for the first time in clinical practice. All approved mRNA vaccines use the lipid‐based nanoparticle delivery system. Several preclinical and clinical trial studies of mRNA vaccines for infectious disease and cancer therapy have also confirmed the reliability of nano‐delivered mRNA‐based therapeutic platforms. For mRNA delivery purposes, nanoparticle should possess several features: effectively encapsulate and protect mRNA from degradation before reaching the target site; and carriers effectively help mRNA delivery into target cells and release mRNA to the lysosome promptly. To this purpose, the commonly used carriers for mRNA delivery, including lipid nanoparticles, LNP, polymer nanoparticles, exosomes, and MVs, are evolved as a promising class of nanomaterials for RNA delivery. We summarized the strengths and weaknesses of various nanomaterials in mRNA delivery (Table 3).

**TABLE 3:** Several advantages and challenges for mRNA delivery

Delivery method	Advantages	Limitations	References
Viral Vectors	Escape the endosome, high transduction efficiency	Large‐scale production, high production costs, potential to induce undesired insertional mutagenesis and immunogenicity	117, 220
Physical methods (microinjection, electroporation, acoustic, laser and magnetic forces)	Low cost, high delivery efficiency	Potential tissue and cellular damage limitations for in vivo use	220, 221
Liposomes	High cargo capacity, escaping phagocytosis	Require complex production, difficult scalability, lack of selectivity for target tissues	48, 222
Lipid nanoparticles	Generally biocompatible, fast and scalability preparation, high reproducibility, high encapsulate efficiency, efficient condensation of mRNA, better kinetic stability	Potential cytotoxicity, relatively short circulation, rapid renal clearance, shorter half‐life blood circulation time, trigger immunogenic response	48
Polymer‐based nanoparticles	Good biocompatibility, easy for fabricate and functionalization, effectively encapsulate and release mRNA, protects RNA from degradation, biodegradable, industrial scale‐up	Safety risks, inherent toxicity of the components, insufficient targeting capabilities, limited efficiency of drug load, possible drug leakage	223
Inorganic nanoparticles	High encapsulation ability, simulate imaging, surface‐engineered, high delivery efficiency, highly stable, improved circulation time	Poor degradability, liver bioaccumulation; low‐tolerability, induce potentially immune responses	224
Lipopolyplex (LPP)	Stability in blood, protectant mRNA from degradation, highly encapsulation efficiency, dendritic cell targeting, endosomal escape	Design and formulation of lipopolyplexes are complex	225, 226
Virus‐like particle (VLP)	Modified for targeted delivery, cost‐effective for large‐scale production, avoid off‐target effects	Unknown immunogenicity, poor stability, extravasate from bloodstream, off‐organ accumulation	166, 169
Virus‐mimicking membrane‐coated nanoparticles	Biosafety, slow elimination, promote the target efficiency	Immunogenicity, unknown escape mechanisms, membrane destabilization	173, 174
Membrane‐coated vehicles	Biocompatible, improve the circulation time, enhance cell encapsulation efficiency	Perturbations in membrane integrity, limit size of the cargo, technologies problem (cell membrane coating, fresh isolation of native cell membranes, co‐extrusion with drug carriers	227
Extracellular vesicle (EVs)	Low‐toxicity, less immunogenicity, biocompatibility, high penetration and retention effect, conveniently cross physiological barriers, intrinsic homing properties, suitable for targeting modification	Heterogeneity of EVs, RNA‐loading capacity is low, low yields, characteristic complexity, lack of access to large‐scale production	216, 228, 229

mRNA must cross several tissue barrier, extracellular barrier, intracellular endosomal escape, and intracellular immunity before it arrives at the intracellular target site. Therefore, it remains challenging to deliver mRNA precisely into the target cells. Most of the existing LNPs are accumulated in the liver after being injected into the body. Therefore, how to precisely delivery sufficient mRNA to target organs when using intramuscular LNP‐RNA is of great importance. This problem can be overcome by selective organ targeting (SORT) strategies. The key to organ‐specific delivery is manipulating the internal or external charges of LNPs. For example, altering the disulfide bonds between the long lipid chains leads to selective accumulation of mRNA formulations in the liver.^230^ While increasing the percentage of permanently positively charged DDAB or EPC can shift tissue tropism from the liver to the lung and introducing the negatively charged SORT molecule 1,2‐dioleoyl‐sn‐glycero‐3‐phosphate (18PA) into LNP can lead to spleen‐specific delivery.^231^ Alternatively, mimetic lipids can be used to synthesize LNP for organ‐targeted delivery. For example, certain neurotransmitters can cross the BBB, and they encapsulate its derived lipidoids to gain access throughout the brain.^232^ Both the modulation of lipid chemistry and nanoparticle fractions allow for organ selectivity, offering the potential for the development of next‐generation mRNA‐targeted delivery vectors.

**FIGURE 6:**
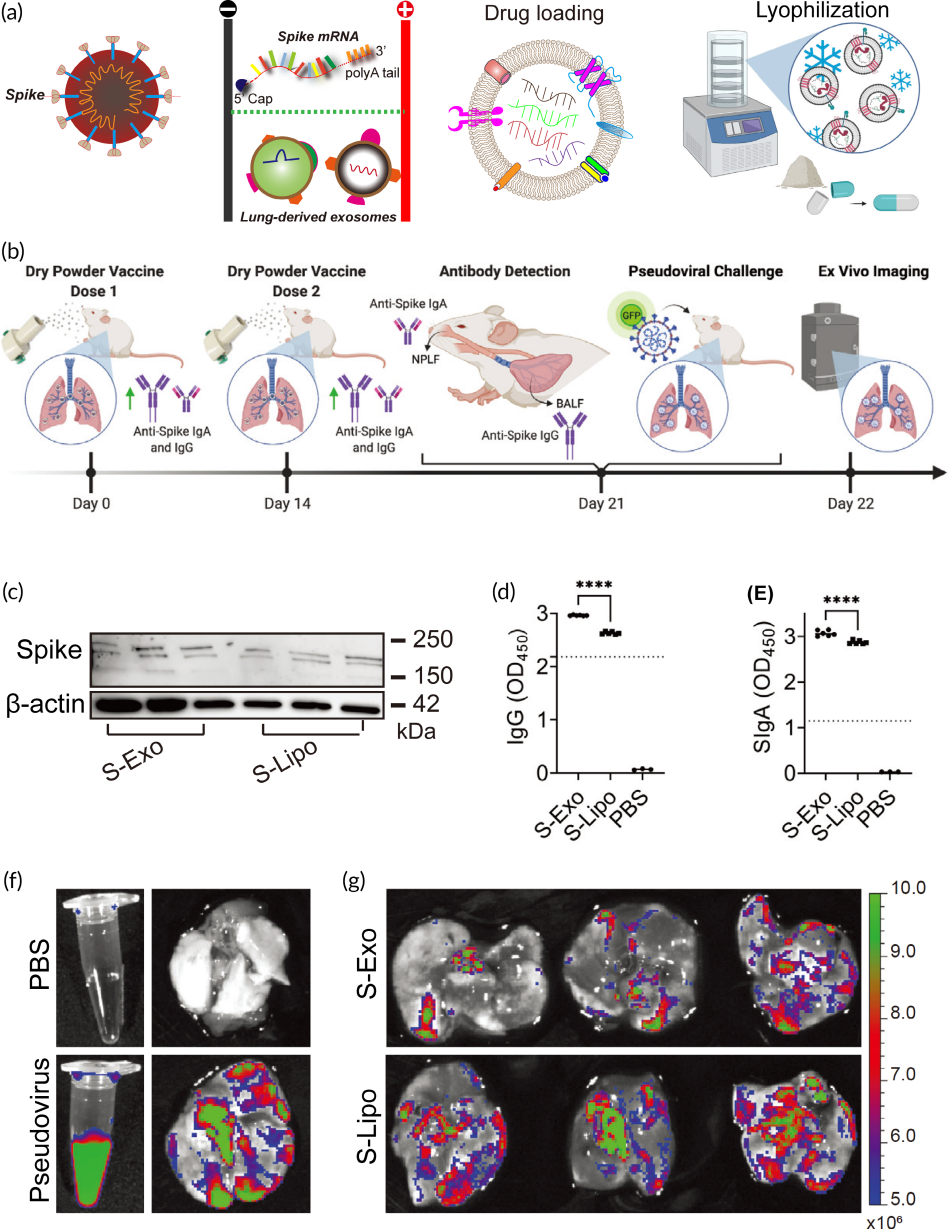
Inhalation of the exosome‐mediated mRNA vaccine elicits immune responses. (a) Pulmonary cell‐derived exosomes are loaded with SARS‐CoV‐2S‐protein mRNA by electro‐transferred and lyophilized to obtain a dry powder formulation. (b) Schematic representation of inhalation process of exosome mRNA, antibody production against S‐protein, and pseudoviral challenge. (c) Identification of spike protein expression in the upper and lower respiratory tracts by western blotting analysis. (d, e) ELISA shows significantly elevated IgG and IgM in both S‐Exos and S‐Lipos. (f) Images of lung tissues from PBS or pseudovirus infection. (g) Biodistribution of the clearance of pseudoviral infection after inhalation of S‐Exo or S‐Lipo vaccine. *Source*: Reproduced with permission.^210^ Copyright 2022, COVID‐19 resource center, Elsevier.

However, the safety of repeated administration of LNPs still remains a challenge for their clinical translation. Exosomes possess great potential as a new type of carrier for mRNA therapy. In particular, exosomes are naturally occurring nanovesicles, making them a safe vehicle for targeted drug delivery. Since exosomes express the surface proteins and receptors of the parent cell, they are advantageous in targeting recipient cells in the same tissue, such as the use of lung spheroid cell‐derived exosome for targeted delivery of vaccines to the lung tissue, as we mentioned in Figure 6. In addition, exosomes can be genetically engineered, chemically surface‐modified, or enzymatically engineered to introduce peptides or proteins to enhance its targeting.^216^, ^228^ Due to their high biocompatibility, bioavailability, and ability to cross biological barriers, there is increasing interest in exosomes as promising carriers for mRNA drug delivery. Exosomes can either encapsulate the transcripted mRNA from parent cells or be directly loaded with synthetic mRNA by conventional transfection methods. In addition, exosomes exhibit great stability in blood, allowing them to transport over long distances in vivo under both physiological and pathological conditions. More importantly, exosomes can be lyophilized as dry powder for inhalation. Accumulating evidence has demonstrated the efficacy and stability of lyophilized lung‐exos at room temperature for up to 28 days.^210^ Current mRNA vaccine products require storage temperatures as low as −20°C or −70°C to ensure stability. Therefore, the use of exosomes as lyophilizable vaccines can achieve a longer shelf life, reduce transport costs, and facilitate vaccine distribution.

One of the leading ongoing challenges for exosomes is the commercially available methods. However, their active substances in exosomes are relatively complex and face a certain degree of inherent biological variation, which may lead to product heterogeneity between different batches. Cell culture is currently one of the main sources of exosomes, while its production and scale are still challenging. In terms of scale‐up production, several strategies have been developed, including the use of bioreactors,^233^ while the cost/yield is still economically unsuitable for clinical application. Alternative sources, including blood, milk, and plant, to obtain exosomes or exosome‐like vesicles may allow for lower production costs and shorter production times.^234^, ^235^ In the future, exosomes are expected to bring breakthroughs in the field of mRNA drugs when technical difficulties are solved.

For the clinical application of nano‐mediated mRNA delivery, it is also necessary to optimize the production and purification methods to obtain a population of suitable size and uniform distribution. In addition, the dose of mRNA delivered by nano‐mediated approaches should also be optimized based on the material. The size, quantity, and loading efficiency should be quantified. At present, most of the research on nanoparticles‐based mRNA therapy remains in the lab bench, further research is needed at the patient's bedside.

## AUTHOR CONTRIBUTIONS

**De‐feng Li:** Conceptualization (lead); writing – original draft (lead); writing – review and editing (lead). **Qi‐song Liu:** Investigation (equal); methodology (equal). **Mei‐feng Yang:** Investigation (equal); methodology (equal). **Hao‐ming Xu:** Data curation (equal); investigation (equal); resources (equal). **Min‐zheng Zhu:** Investigation (equal); methodology (equal). **Yuan Zhang:** Resources (equal); validation (equal). **Jing Xu:** Project administration (equal); supervision (equal); visualization (equal). **Cheng‐mei Tian:** Data curation (equal); formal analysis (equal). **Jun Yao:** Supervision (equal); validation (equal); writing – review and editing (equal). **Li‐sheng Wang:** Project administration (equal); supervision (equal); writing – review and editing (equal). **Yu‐jie Liang:** Conceptualization (equal); funding acquisition (equal); supervision (equal); writing – review and editing (equal).

## FUNDING INFORMATION

This work was funded by the Science and Technology Innovation Committee of Shenzhen (No. JCYJ20200109150700942, No. CXFZ20211020164543006), Shenzhen Clinical Research Center for Mental Disorders (20210617155253001), the Guangdong Basic and Applied Basic Research Foundation (2020A1515011581), the Key‐Area Research and Development Program of Guangdong Province (2019B030335001), the Shenzhen Fund for Guangdong Provincial High Level Clinical Key Specialties (No. SZGSP013), the Shenzhen Key Medical Discipline Construction Fund (No. SZXK042), and  Shenzhen Science and Technology Projects (No. KCXFZ20211020164543006, No. JCYJ20150403101028164, No. JCYC20170307100911479, No. JCYJ20190807145617113 and JCYJ20210324113802006).

## CONFLICT OF INTEREST

The authors declare no competing interests.

### PEER REVIEW

The peer review history for this article is available at https://publons.com/publon/10.1002/btm2.10492.

## Data Availability

There are no supplementary data available for this article.
